# Seldinger method for intraoperative cholangiography: *a* practical approach

**DOI:** 10.1308/003588412X13171221591259b

**Published:** 2012-05

**Authors:** N Abbassi-Ghadi, N Menezes

**Affiliations:** St Peter–s Hospital, ChertseyUK

## BACKGROUND

Manipulation of a catheter Into the cystic duct for successful intraoperative cholangiography can be made difficult by the tortuosity of the cystic duct and the spiral valves of Heister.^1^ We describe a technique to eliminate these problems and allow successful positioning of the catheter within the cystic duct.

## TECHNIQUE

**Figure 1:**
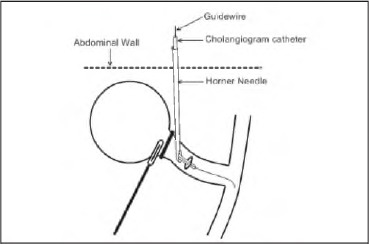
Position of cholangiogram catheter and needle

A Steriseal Horner needle (Optech Diagnostic & Surgical, East Melbourne, Australia) Is guided through the abdominal wall and placed adjacent to the incision created In the cystic duct. The cholangiogram catheter Is passed through the needle to protrude through Its laterally placed end opening (Fig 1). This opening angulates the catheter tip, allowing It to be easily negotiated Into the cystic duct.

If the catheter cannot be manipulated into an adequate position within the duct, a Zebra® straight tip guldewire (0.035ln x 150cm, Boston Scientific, Miami, FL, US) can be fed through the catheter and passed further into the duct. The position of the wire can be checked with an x-ray Image intensifier. The catheter can then be passed over the top of the guldewire In a Seldinger fashion. Once in the correct position, It is secured with a clip, and the guidewlre and Horner needle are removed successively.

## DISCUSSION

The advantage of the guidewire is that it readily passes through a tortuous duct or the valves of Heister without difficulty and allows the cholangiogram catheter to slide over it with ease. Ultimately, this Seldinger method should save time, avoiding multiple attempts at manipulating the catheter into the correct position within the cyst duct.
